# Regulation of Guinea Pig Detrusor Smooth Muscle Excitability by 17β-Estradiol: The Role of the Large Conductance Voltage- and Ca^2+^-Activated K^+^ Channels

**DOI:** 10.1371/journal.pone.0141950

**Published:** 2015-11-04

**Authors:** Aaron Provence, Kiril L. Hristov, Shankar P. Parajuli, Georgi V. Petkov

**Affiliations:** Department of Drug Discovery and Biomedical Sciences, South Carolina College of Pharmacy, University of South Carolina, Columbia, South Carolina, United States of America; Indiana University School of Medicine, UNITED STATES

## Abstract

Estrogen replacement therapies have been suggested to be beneficial in alleviating symptoms of overactive bladder. However, the precise regulatory mechanisms of estrogen in urinary bladder smooth muscle (UBSM) at the cellular level remain unknown. Large conductance voltage- and Ca^2+^-activated K^+^ (BK) channels, which are key regulators of UBSM function, are suggested to be non-genomic targets of estrogens. This study provides an electrophysiological investigation into the role of UBSM BK channels as direct targets for 17β-estradiol, the principle estrogen in human circulation. Single BK channel recordings on inside-out excised membrane patches and perforated whole cell patch-clamp were applied in combination with the BK channel selective inhibitor paxilline to elucidate the mechanism of regulation of BK channel activity by 17β-estradiol in freshly-isolated guinea pig UBSM cells. 17β-Estradiol (100 nM) significantly increased the amplitude of depolarization-induced whole cell steady-state BK currents and the frequency of spontaneous transient BK currents in freshly-isolated UBSM cells. The increase in whole cell BK currents by 17β-estradiol was eliminated upon blocking BK channels with paxilline. 17β-Estradiol (100 nM) significantly increased (~3-fold) the single BK channel open probability, indicating direct 17β-estradiol-BK channel interactions. 17β-Estradiol (100 nM) caused a significant hyperpolarization of the membrane potential of UBSM cells, and this hyperpolarization was reversed by blocking the BK channels with paxilline. 17β-Estradiol (100 nM) had no effects on L-type voltage-gated Ca^2+^ channel currents recorded under perforated patch-clamp conditions. This study reveals a new regulatory mechanism in the urinary bladder whereby BK channels are directly activated by 17β-estradiol to reduce UBSM cell excitability.

## Introduction

The functions of the urinary bladder, which are to store and periodically release urine, are facilitated by the contraction and relaxation of urinary bladder smooth muscle (UBSM). Overactive bladder (OAB), a highly prevalent chronic health condition in the United States, is often associated with increased UBSM contractility. The primary treatment for OAB involves antimuscarinic agents, which have limited efficacy and tolerability [1]. Many forms of OAB have been linked directly to UBSM dysfunction [1, 2]. Therefore, novel therapeutic modalities for OAB, targeting UBSM directly, are urgently needed.

Sharing a common embryonic origin, the genital and lower urinary tract systems are both regulated by sex hormones, including estrogens [3, 4]. Systemic and vaginal estrogen therapies have been considered beneficial in alleviating symptoms of OAB in postmenopausal women [3, 4]. Epidemiological studies have also linked post-menopausal estrogen deficiencies with the increased risk for OAB [3]. Despite these observations, conflicting evidence in the literature exists concerning the role of estrogen as a treatment for OAB [3]. Some studies suggest beneficial effects of estrogen replacement therapies for controlling symptoms of OAB, while other studies report the opposite [3–5]. Thus, there remains the need for an improved understanding of the mechanisms by which estrogens regulate UBSM function [5].

The predominant estrogen in human circulation is 17β-estradiol, a potent hormone known to regulate urinary bladder function [3, 4, 6, 7]. 17β-Estradiol-induced UBSM relaxation has long been established *in vitro* [8–10] and *in vivo* [11]. The cellular mechanisms of these functional effects of 17β-estradiol in UBSM are not well understood, but several mechanisms have been suggested, including L-type voltage-gated Ca^2+^ (Ca_V_) channel inhibition [2, 9] and K^+^ channel activation [12]. Among the K^+^ channel targets of 17β-estradiol are the large conductance voltage- and Ca^2+^-activated K^+^ (BK) channels [13–16]. A previous study [12] suggested the possible involvement of the BK channels in 17β-estradiol-induced UBSM relaxation as the relaxant effects of 17β-estradiol were concentration-dependently blocked by the specific BK channel inhibitor iberiotoxin. However, the role of the BK channels as targets for 17β-estradiol has never investigated at the cellular level in UBSM.

BK channels are among the most physiologically-relevant K^+^ channels regulating UBSM excitability and contractility [17, 18]. As both a Ca^2+^ and voltage sensor, BK channels work to oppose UBSM excitability by promoting cell membrane hyperpolarization, which in turn precludes Ca^2+^ influx through L-type Ca_V_ channels to promote UBSM relaxation [17, 18]. In UBSM, BK channels are activated by either Ca^2+^ influx through L-type Ca_V_ channels or by Ca^2+^ released from the sarcoplasmic reticulum (SR) ryanodine receptors (RyRs), known as “Ca^2+^ sparks” [18]. BK channel activation by Ca^2+^ sparks triggers transient BK currents (TBKCs), also known as spontaneous transient outward currents (STOCs) [18], which regulate UBSM excitability. Given the key role of the BK channels in UBSM function [17, 18], their potential regulation by 17β-estradiol represents a critically important mechanism in UBSM cell physiology. Indeed, an earlier study proposed a potential role for the BK channels in 17β-estradiol-mediated UBSM relaxation [12]. However, in UBSM, the existence of a 17β-estradiol-BK channel functional interaction has not been investigated at the cellular level. Therefore, this study aimed to elucidate the functional role of the BK channels as non-genomic targets of 17β-estradiol in guinea pig UBSM cell excitability. We employed multiple electrophysiological protocols including single BK channel recordings on inside-out excised membrane patches and the amphotericin-B perforated whole cell patch-clamp technique in combination with the selective BK channel inhibitor paxilline.

## Materials and Methods

### UBSM tissue acquisition and single cell isolation

All experimental procedures were conducted in accordance with the animal use protocol #2186 reviewed and approved by the Institutional Animal Care and Use Committee (IACUC) of the University of South Carolina. Only male animals were used in this study to avoid changes in the estrogen levels that occur during the menstrual cycle of the females. Forty-six adult male Hartley-Albino guinea pigs (Charles River Laboratories, Raleigh, NC) of average weight 740±177 g were euthanized by CO_2_ inhalation using a SMARTBOX^™^ automated CO_2_ delivery system (Euthanex Corp, Palmer, PA) followed by thoracotomy. Subsequently, the urinary bladder was removed after a transverse incision superior to the bladder neck. Dissection of UBSM tissues was performed as previously described [19]. UBSM single cells were isolated from UBSM tissues by enzymatic digestion using a combination of collagenase and papain as previously described [19]. Freshly-isolated UBSM cells were used for patch-clamp experiments within 12 h of isolation.

### Patch-clamp electrophysiology

UBSM cell suspension (0.3–0.5 ml) was placed in a glass-bottom chamber to settle for at least 20–30 min. We applied the amphotericin-B perforated whole cell patch-clamp technique as previously described [19] to record voltage-step depolarization-induced whole cell BK currents, TBKCs, L-type Ca_V_ currents, and the resting membrane potential of freshly-isolated guinea pig UBSM cells. To determine the effects of 17β-estradiol on whole cell steady-state BK currents, UBSM cells were voltage-clamped at a holding potential of -70 mV. Subsequently, voltage-step depolarizations were applied from -40 to +80 mV at 20 mV intervals for 200 ms. The threshold of STOCs was set at three times the single BK channel amplitude at -20 mV, or at 9 pA. Membrane potential recordings were performed in current-clamp mode (*I = 0*) of the patch-clamp technique. The effects of 17β-estradiol on peak L-type Ca_V_ channel currents were recorded at 0 mV in voltage-clamp mode of the amphotericin-B perforated patch-clamp technique in the presence of paxilline (1 μM). Single BK channel recordings were performed on inside-out excised membrane patches as previously described [19]. Single BK channel currents were measured at -60 mV with bath and pipette solutions containing symmetrical 140 mM KCl and ~300 nM free [Ca^2+^] (see **§Solutions and drugs**). These experiments were conducted using pCLAMP version 10.2 software (Molecular Devices, Sunnyvale, CA) with an Axopatch 200B amplifier (Digidata 1322A). Currents were filtered using an eight-pole Bessel filter (model 900CT/9L8L, Frequency Devices, Ottawa, IL). Borosilicate glass patch-clamp pipettes (Sutter Instruments, Novato, CA) were pulled using a Narishige glass micropipette puller (model PP-830, Narishige Group, Tokyo, Japan) and polished with a Microforge (model MF-830, Narishige Group). The final pipette resistance was 4–6 MΩ for whole cell patch-clamp and 6–15 MΩ for single BK channel recordings. All patch-clamp experiments were conducted at room temperature (22–23°C).

### Solutions and drugs

Ca^2+^-free dissection solution contained (in mM): 80 monosodium glutamate; 55 NaCl; 6 KCl; 10 glucose; 10 N-2-hydroxyethylpiperazine-N'-2-ethanesulphonic acid (HEPES); 2 MgCl_2_; NaOH was administered to attain pH 7.3. The extracellular solution for whole cell patch-clamp experiments had (in mM): 134 NaCl; 6 KCl, 1 MgCl_2_, 2 CaCl_2_, 10 glucose, and 10 HEPES, pH adjusted to 7.4 with NaOH. The patch-pipette solution contained (in mM): 110 potassium aspartate; 30 KCl; 10 NaCl; 1 MgCl_2_; 10 HEPES; 0.05 ethylene glycol-bis(2-aminoethylether)-N,N,N',N'-tetraacetic acid (EGTA); NaOH was used to adjust the pH to 7.2. Symmetrical K^+^ solution used for single BK channel recordings contained (in mM): 140 KCl; 1.08 MgCl_2_; 5 EGTA, and 3.16 CaCl_2_, adjusted to pH 7.2 with NaOH. Stock amphotericin-B solution was freshly prepared daily in dimethyl sulfoxide (DMSO) and was added to the pipette solution (200–300 μg/ml) prior to the experiment. 17β-Estradiol and paxilline were purchased from Sigma-Aldrich (St. Louis, MO) and were dissolved in DMSO. The final concentration of DMSO in the bath solution did not exceed 0.01%.

### Data analysis and statistics

Single BK channel openings were analyzed over 5–10 min intervals before and after the addition of 17β-estradiol (100 nM). The values for single BK channel open probability [4] of each excised patch (*NP*_*o*_) were calculated using Clampfit 10.2 software as previously described [19]. Single BK channel *P*_*o*_ for each patch was calculated as *NP*_*o*_ where *N* refers to the number of channels in the patch. The effects of 17β-estradiol on whole cell steady-state BK currents and the cell membrane potential were analyzed using Clampfit 10.2 software. The effects of 17β-estradiol on voltage-step depolarization-induced whole cell BK currents were analyzed by taking the average value of the last 50 ms of each pulse before and after the application of 17β-estradiol (100 nM). The effects of 17β-estradiol on the amplitude and frequency of TBKCs were analyzed using Minianalysis software (Synaptosoft, Decatur, GA). Data are presented as the means ± SEM. In the summarized data, “**n**” indicates the number of UBSM cells used and “**N**” represents the total number of guinea pigs. CorelDraw Graphics Suite X3 software (Corel Co., Mountain View, CA) and GraphPad Prism 4.03 software (GraphPad Software, Inc., La Jolla, CA) were used for statistical analysis and data illustration. Two-way ANOVA followed by Bonferroni post-hoc test were performed to evaluate the effects of 17β-estradiol on whole cell BK currents. Paired Student’s t-test was used for all other experimental series. P values <0.05 were considered statistically significant.

## Results

### 17β-Estradiol enhanced whole cell depolarization-induced steady-state K^+^ currents in freshly-isolated UBSM cells

**Fig 1:**
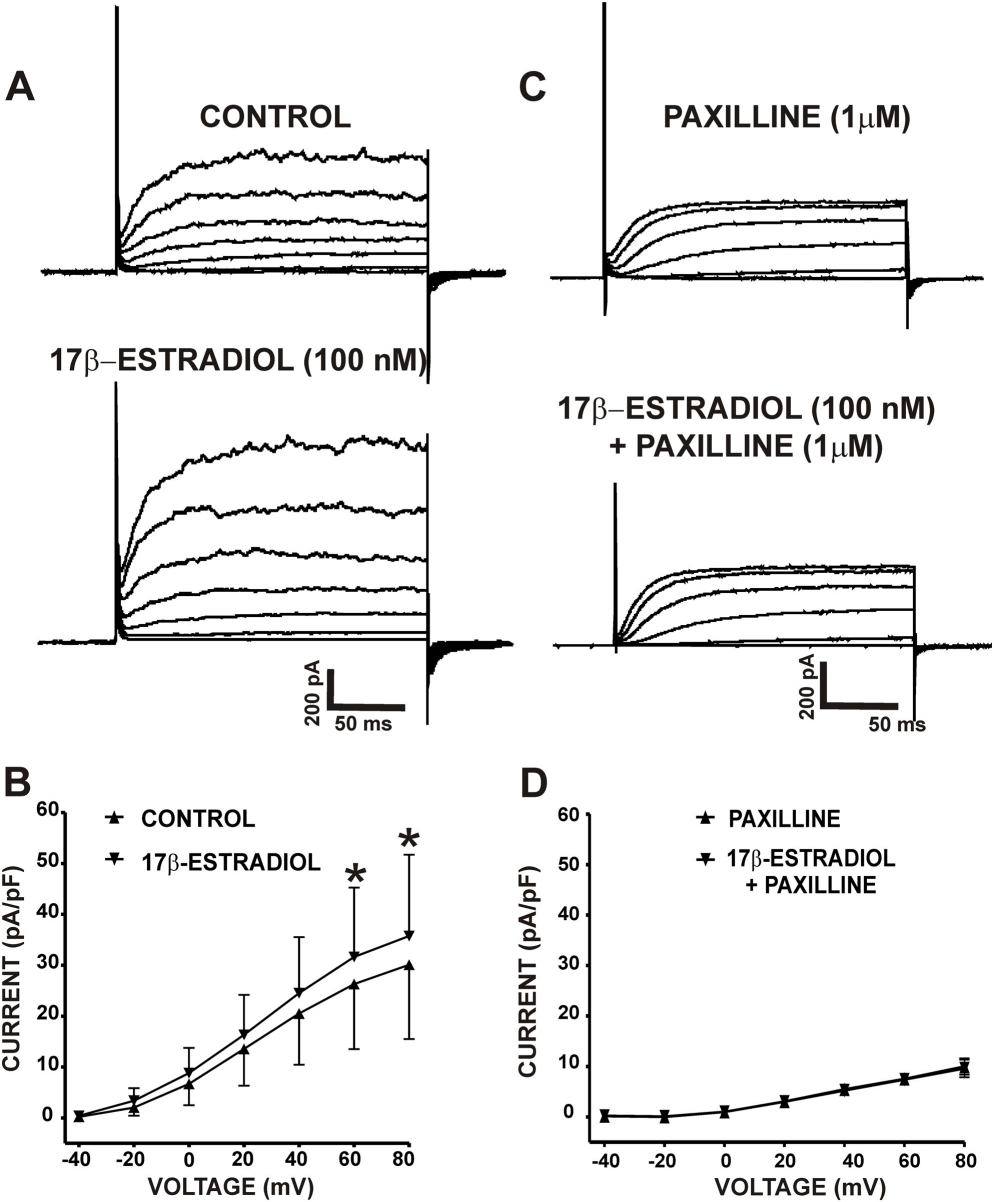
17β-Estradiol increases depolarization-induced whole cell steady-state BK currents in freshly-isolated UBSM cells. **A)** Representative traces from a voltage-clamp experiment illustrating that 17β-estradiol (100 nM) increases whole cell steady-state BK currents in an isolated UBSM cell. **B)** The current-voltage relationship curves illustrate the 17β-estradiol (100 nM)-mediated increase of whole cell steady-state BK currents in isolated UBSM cells (n = 11, N = 10; *P<0.05). **C)** Representative traces from a voltage-clamp experiment illustrating the lack of effects of 17β-estradiol (100 nM) on whole cell steady-state BK currents in the presence of the BK channel inhibitor paxilline (1 μM). **D)** The current-voltage relationship curves illustrate that 17β-estradiol (100 nM) had no significant effects on whole cell steady-state BK currents in the presence of 1 μM paxilline (n = 14, N = 12; P>0.05).

The average whole cell capacitance of all cells used in the present study was 29.8±2.2 pF (n = 64, N = 46). 17β-Estradiol (100 nM) caused a significant increase in whole cell steady-state K^+^ currents (n = 11, N = 10; P<0.05, Fig 1A and 1B). As illustrated in Fig 1A and 1B, the density of whole cell steady-state K^+^ currents at +80 mV were 30.1±14.6 pA/pF for the controls and 35.8±16.0 pA/pF in the presence of 17β-estradiol. To determine whether these stimulatory effects were mediated by the BK channels, we examined the effects of 17β-estradiol in the presence of the BK channel inhibitor paxilline (1 μM). As shown in Fig 1C and 1D, when BK channels were blocked with 1 μM paxilline, 17β-estradiol had no significant effects on the residual whole cell steady-state K^+^ currents. The density of whole cell steady-state K^+^ currents at +80 mV was 13.5±2.3 pA/pF for the controls (paxilline only) and after the application of 17β-estradiol in the continued presence of paxilline was 13.4±2.4 pA/pF (n = 14, N = 12; P>0.05; Fig 1C and 1D). These results indicate that in UBSM cells, the increase in whole cell steady-state K^+^ currents by 17β-estradiol is mediated by the BK channels.

### 17β-Estradiol increased the frequency of TBKCs in freshly-isolated UBSM cells

**Fig 2:**
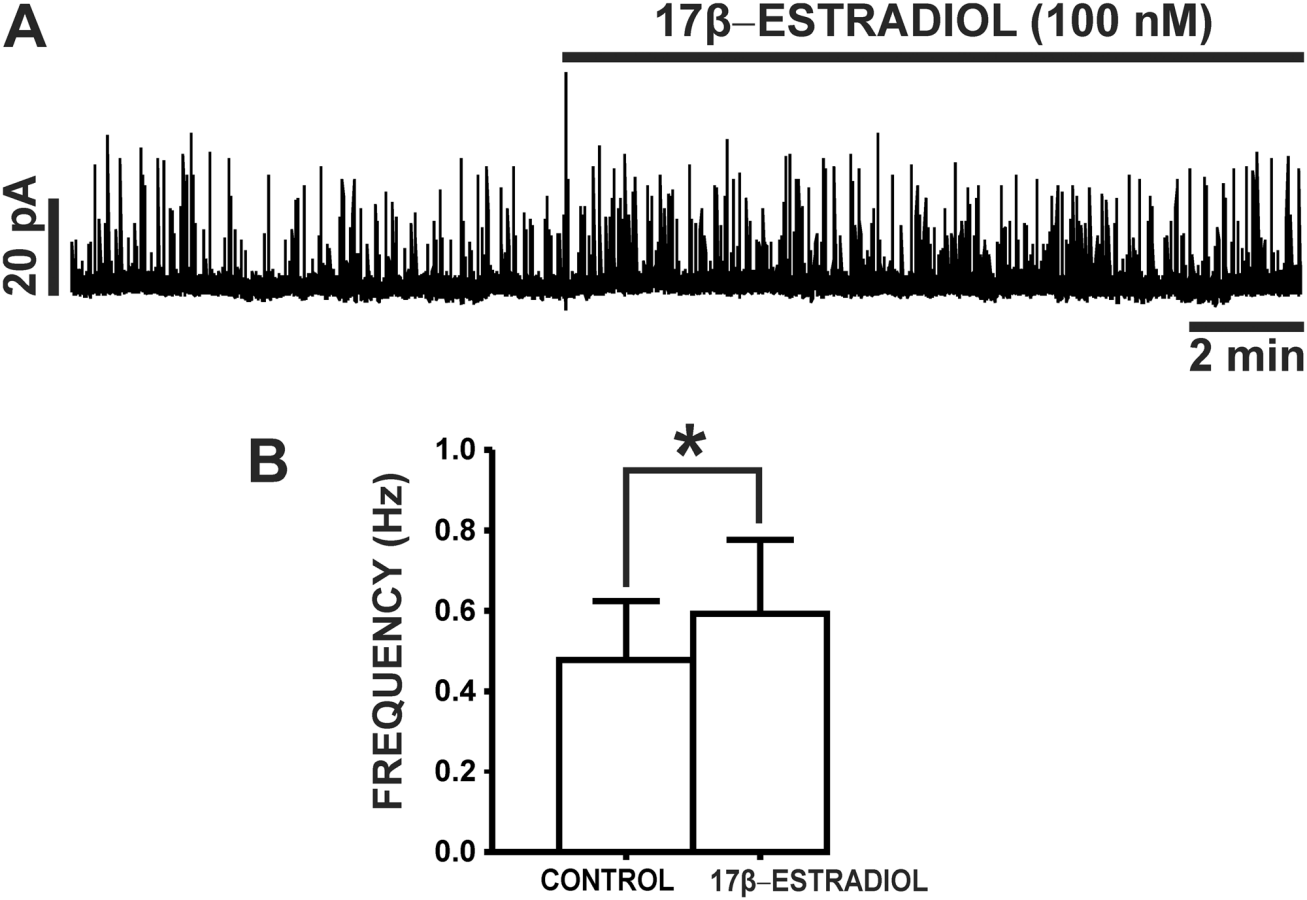
17β-Estradiol increases the frequency of TBKCs in freshly-isolated UBSM cells. **A)** An original representative recording illustrating the stimulatory effects of 17β-estradiol (100 nM) on TBKC activity in an isolated UBSM cell. **B)** Summarized data illustrating the increase in TBKC frequency by 17β-estradiol (100 nM) in UBSM cells (n = 9, N = 9; *P<0.05).

TBKCs have a key role in regulating UBSM cell excitability [17, 18]. We sought to elucidate the regulation of TBKC activity by 17β-estradiol in UBSM cells. TBKCs were recorded at a holding potential of -20 mV. As illustrated in Fig 2, 17β-estradiol (100 nM) significantly increased the frequency of TBKCs from 0.48±0.15 Hz (control) to 0.59±0.18 Hz (n = 9, N = 9; P<0.05), without effecting TBKC amplitude (n = 9, N = 9; P>0.05). Collectively, these data indicate that 17β-estradiol regulates TBKC activity in UBSM cells.

### 17β-Estradiol increased single BK channel *P*_*o*_ in UBSM cell excised membrane patches

**Fig 3:**
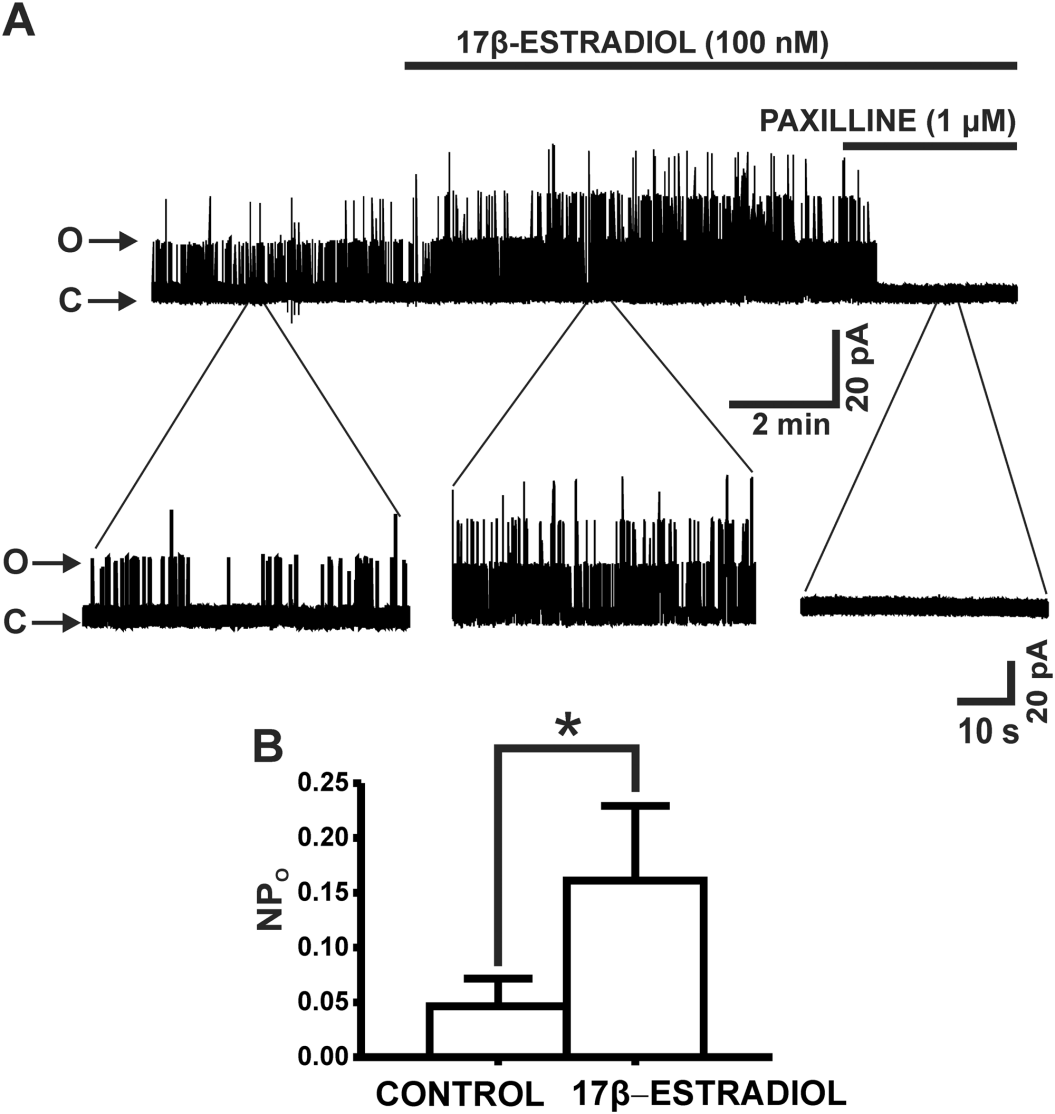
17β-Estradiol increases single BK channel *P*_*o*_ in excised patches from freshly-isolated UBSM cells. **A)** An original representative recording of BK channel currents recorded in the inside-out configuration of the patch-clamp technique before and after the addition of 100 nM 17β-estradiol in an excised patch from an isolated UBSM cell. Single channel activity was completely abolished by 1 μM paxilline. “**O**” indicates open channel state and “**C**” indicates closed channel state. **B)** Summarized data for the effects of 17β-estradiol (100 nM) on the BK channel *NP*_*o*_ (n = 13, N = 11; *P<0.05). Single BK channel recordings were performed at a holding potential of -60 mV.

To elucidate if 17β-estradiol has a direct effect on the BK channels, we conducted single BK channel recordings on inside-out excised membrane patches. 17β-Estradiol (100 nM) increased the mean BK channel *NP*_*o*_ from the control value of 0.05±0.03 to 0.20±0.07 (n = 13, N = 11; P<0.05). Further, as illustrated in Fig 3, single BK channel opening events were completely abolished by 1 μM paxilline in all excised membrane patches. These data provide strong support that 17β-estradiol directly and rapidly activates BK channels in UBSM cells.

### 17β-Estradiol hyperpolarized the resting membrane potential of UBSM cells

**Fig 4:**
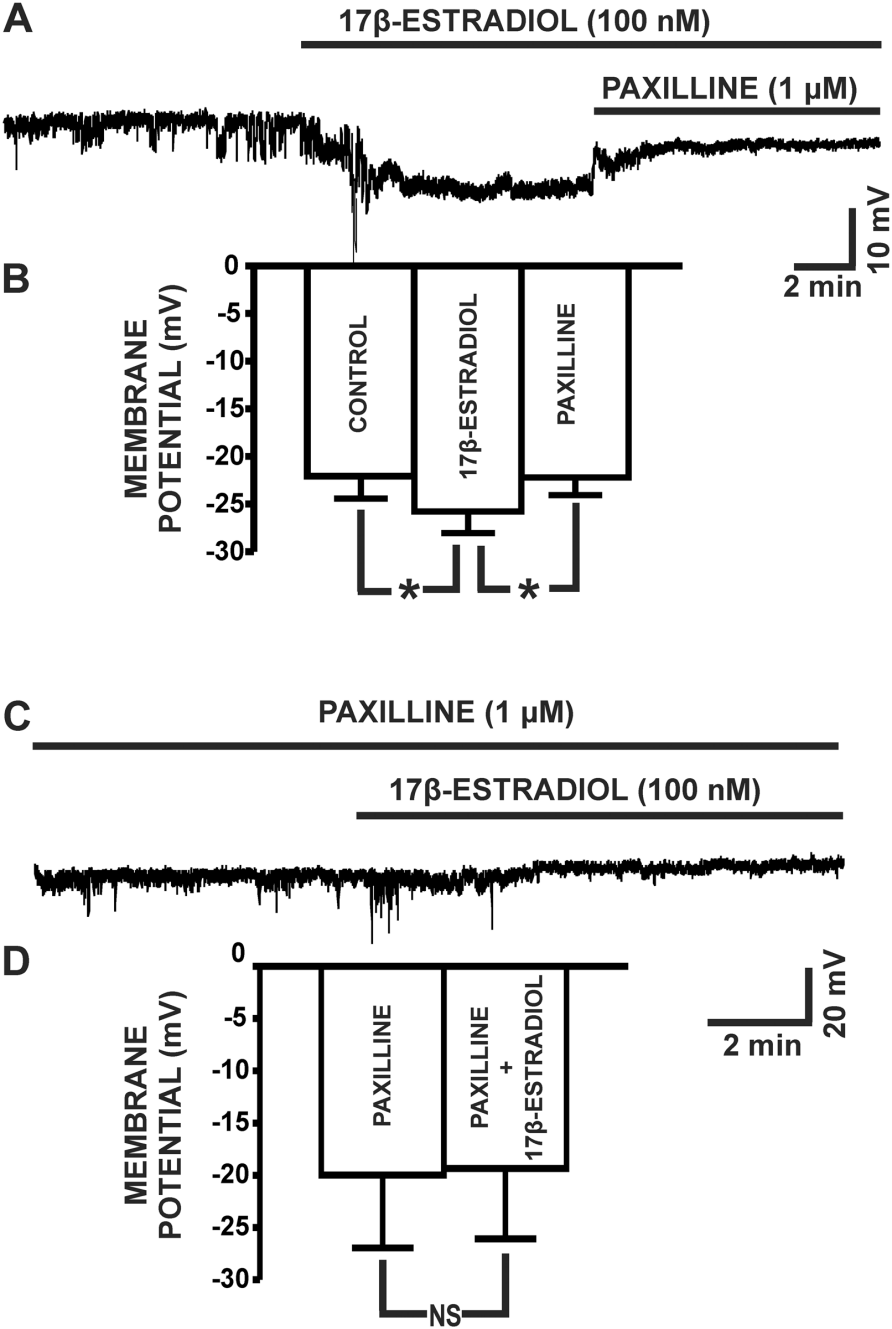
17β-Estradiol hyperpolarizes the membrane potential of freshly-isolated UBSM cells. **A)** A representative trace of a membrane potential recording in current-clamp mode (*I = 0*) demonstrating the hyperpolarizing effects of 17β-estradiol (100 nM) in an isolated UBSM cell. The hyperpolarizing effect of 17β-estradiol (100 nM) was reversed by 1 μM paxilline. **B)** Summarized data illustrating the hyperpolarizing effects of 17β-estradiol on the UBSM cell membrane potential and that 1 μM paxilline reverses the 17β-estradiol-induced hyperpolarization (n = 12, N = 11; *P<0.05). **C)** A representative trace of a membrane potential recording in current-clamp mode demonstrating that when the BK channels are blocked with 1 μM paxilline, 17β-estradiol (100 nM) did not cause membrane hyperpolarization in an isolated UBSM cell. **D)** Summarized data illustrating that 17β-estradiol had no effect on the UBSM cell membrane potential in the presence of 1 μM paxilline (n = 7, N = 7; P>0.05).

Next, we aimed to elucidate the BK channel-dependent regulation of the UBSM cell membrane potential by 17β-estradiol in UBSM cells. 17β-Estradiol (100 nM) significantly hyperpolarized the UBSM cell membrane potential to -25.8±2.3 mV from the control value of -22.1±2.4 mV (n = 12, N = 11; P<0.05; Fig 4A and 4B). The BK channel inhibitor paxilline (1 μM) reversed the hyperpolarizing effects of 17β-estradiol on the UBSM cell membrane potential to -22.2±1.9 mV (n = 12, N = 11; P<0.05; Fig 4A and 4B). As shown in Fig 4C and 4D, 17β-estradiol had no effects on the UBSM cell membrane potential when administered in the presence of paxilline (1 μM), with the membrane potential of -19.3±6.7 mV in comparison to the control (paxilline only) value of -20.0±7.0 mV (n = 7, N = 7; P>0.05). These data support the concept that 17β-estradiol regulates the UBSM cell membrane potential through a mechanism involving the modulation of BK channel activity.

### 17β-Estradiol did not inhibit L-type Ca_V_ currents in freshly-isolated UBSM cells

**Fig 5:**
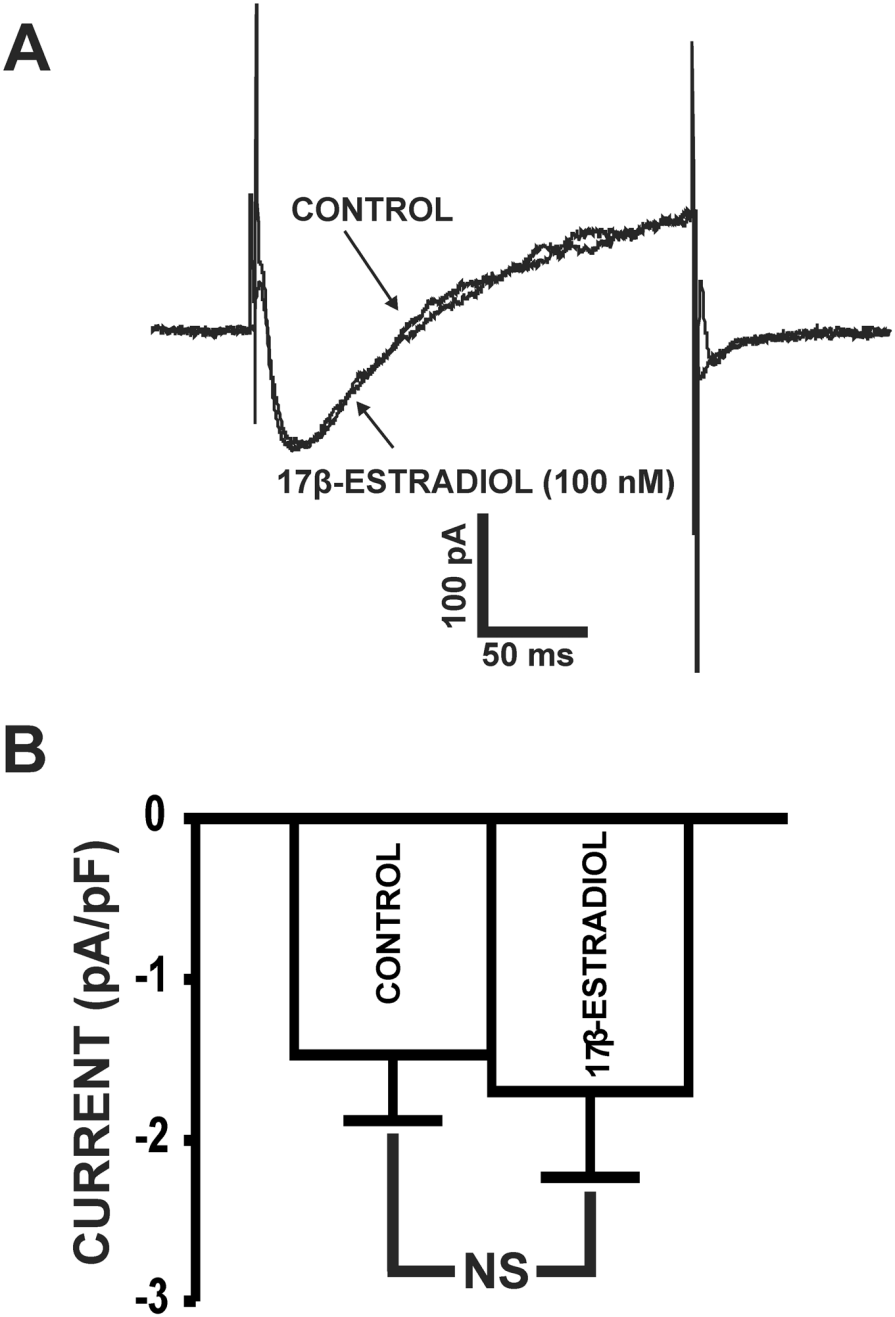
17β-Estradiol does not inhibit L-type Ca_V_ channel activity in freshly-isolated UBSM cells. **A)** A representative recording of the peak L-type Ca_V_ channel currents recorded at 0 mV in a freshly-isolated UBSM cell in the absence (control) or presence of 17β-estradiol (100 nM). **B)** Summary data of the current density of L-type Ca_V_ channel currents in the absence (control) or presence of 100 nM 17β-estradiol (n = 5, N = 5; P>0.05).

In guinea pig UBSM cells, the inhibition of L-type Ca_V_ currents by 17β-estradiol has been reported at micromolar concentrations using the conventional patch-clamp technique [9], which does not maintain the native physiological environment of UBSM cells. Thus, we sought to determine whether nanomolar concentrations of 17β-estradiol (100 nM) modulates L-type Ca_V_ channel activity in UBSM cells in the presence of the BK channel inhibitor paxilline (1 μM) using the perforated patch-clamp technique. As exemplified by Fig 5, 17β-estradiol (100 nM) had no effect on L-type Ca_V_ channel currents. At 0 mV, the average inward current density under control conditions was -1.4±0.3 pA/pF and after the addition of 100 nM 17β-estradiol was -1.6±0.4 pA/pF (n = 5, N = 5; P>0.05; Fig 5). These results support the novel concept that under physiological conditions of the perforated patch-clamp, nanomolar concentrations of 17β-estradiol do not directly affect L-type Ca_V_ channel activity.

## Discussion

The current study provided the first electrophysiological evidence establishing the novel regulatory mechanism by which the BK channels are direct targets for 17β-estradiol at nanomolar concentrations in guinea pig UBSM cells. Our results demonstrate that 17β-estradiol rapidly increases: 1) the amplitude of whole cell steady-state BK currents; 2) the frequency of TBKCs; 3) single BK channel *NP*_*o*_; and 4) hyperpolarizes the UBSM cell membrane potential; and 5) does not directly inhibit L-type Ca_V_ channel currents at nanomolar concentrations.

17β-Estradiol is most widely known for its long-term genomic mechanisms involving gene expression and the promotion of female sexual and reproductive health. However, emerging evidence in UBSM and other cell types suggests the existence of several non-genomic mechanisms of 17β-estradiol, which may acutely influence cell excitability and contractility. These non-genomic mechanisms include: inhibition of L-type Ca_V_ channels [9, 20, 21], endothelial-dependent release of nitric oxide [22], activation of protein kinases [23], and activation of the BK channels [13, 24]. Involvement of the BK channels in the mechanism of 17β-estradiol-induced response is supported by studies from non-UBSM cell types and recombinant systems expressing BK channels, which indicate the activation of BK channels by 17β-estradiol is dependent on their regulatory β1-subunit [5, 13–16]. The role of the regulatory β1-subunit was also supported by studies from freshly-isolated murine colonic myocytes, where the increase in BK channel *P*_*o*_ by 17β-estradiol was not observed in excised membrane patches from β1-subunit knockout (β1^-/-^) mice [25].

In UBSM, *in vitro* functional studies have demonstrated the inhibition of rodent and pig UBSM contractility by 17β-estradiol [8, 10, 12]. These observations correspond to reports in rat UBSM, where 17β-estradiol decreased the amplitude and frequency of spontaneous Ca^2+^ flashes [10]. A previous study in guinea pigs showed that 17β-estradiol-induced inhibition of UBSM contractility was still achieved in the presence of the estrogen receptor antagonist ICI-182,780 [10]. This finding supports a non-genomic mechanism for 17β-estradiol in controlling UBSM contractility that is independent of estrogen receptor activation. In guinea pig UBSM, it has been suggested that 17β-estradiol acts as both an L-type Ca_V_ channel inhibitor and K^+^ channel activator [12]. It has been shown that micromolar concentrations of 17β-estradiol inhibit L-type Ca^2+^ currents in guinea pig UBSM cells [9], while a separate study showed the relaxant effects of 17β-estradiol on guinea pig UBSM contractility were blocked in a concentration-dependent manner by the selective BK channel inhibitor iberiotoxin [12]. As BK and L-type Ca_V_ channels are functionally coupled [17, 18], it was important to elucidate these pathways at the cellular level in UBSM using nanomolar concentrations of 17β-estradiol under physiological experimental conditions.

Our results show that 17β-estradiol increased the amplitude of whole cell steady-state K^+^ currents (Fig 1) consistent with reports in non-UBSM cell types [14, 25]. The rapid effects of 17β-estradiol on whole cell K^+^ currents were blocked by the BK channel inhibitor paxilline, suggesting a BK channel-dependent mechanism. In UBSM cells, Ca^2+^ sparks released from the SR RyRs activate TBKCs, which fundamentally regulate UBSM cell excitability [17, 18]. Activation of TBKCs hyperpolarizes the UBSM cell membrane potential and decreases Ca^2+^ influx through L-type Ca_V_ channels [17, 18]. 17β-Estradiol (100 nM) caused a significant increase in the frequency of TBKCs **(** Fig 2 **)**, indicating that 17β-estradiol positively modulates BK channel activity in UBSM cells.

Directly confirming the 17β-estradiol-BK channel functional interactions, we found that 17β-estradiol (100 nM) significantly increased the single BK channel *NP*_*o*_ of inside-out excised membrane patches of UBSM cells (Fig 3). These results are in support of earlier electrophysiological reports in non-UBSM cell types showing activation of the BK channels by 17β-estradiol [13–16, 26]. Our single BK channel recordings were performed using symmetrical K^+^ solutions with fixed Ca^2+^ concentration and in the absence of signaling pathways that may alter BK channel P_o_. The rapid effects of 17β-estradiol on the UBSM BK channels further indicate direct non-genomic mechanisms. Thus, our data provide clear-cut evidence that 17β-estradiol directly activates BK channels in UBSM cells.

The negative feedback mechanism by which BK channels limit UBSM excitability originates from its ability to control the cell membrane potential [18]. 17β-Estradiol (100 nM) caused a BK channel-dependent cell membrane hyperpolarization in UBSM cells (Fig 4). 17β-Estradiol-mediated hyperpolarization in UBSM cells is consistent with the stimulatory effects of 17β-estradiol on TBKCs and whole cell steady-state BK currents.

Our study is the first to reveal a direct functional role for the BK channels in mediating the effects of 17β-estradiol in UBSM at the cellular level. Importantly, the effects of 17β-estradiol on BK channel activity in the current study were achieved using a nanomolar concentration, well below the micromolar concentrations previously used in UBSM functional studies [8–10, 12]. In particular, it was previously reported using the conventional patch-clamp technique that 17β-estradiol, at much higher non-physiological micromolar concentrations (1 μM), inhibited peak Ca^2+^ currents by 50% in guinea pig UBSM cells [9]. However, 17β-estradiol never reaches micromolar concentrations in the blood plasma even under pathophysiological conditions. We demonstrated here for the first time using the perforated whole cell patch-clamp technique, that 17β-estradiol (100 nM) had no direct effects on L-type Ca_V_ channel activity in freshly-isolated guinea pig UBSM cells (Fig 5). These data suggest that 17β-estradiol directly activates the BK channels at nanomolar concentrations, which are insufficient to affect L-type Ca_V_ channel activity. Unlike our study, previous studies that showed L-type Ca_V_ channel inhibition by 17β-estradiol in guinea pig UBSM cells have used physiologically-irrelevant high micromolar concentrations [9]. Thus, our study reveals the novel findings that L-type Ca_V_ channel inhibition by 17β-estradiol is not involved in UBSM relaxation under normal physiological conditions.

The current study provides strong evidence supporting a role for the BK channels as low-affinity non-genomic targets for 17β-estradiol in UBSM. Our study does not exclude the possibility that 17β-estradiol may exert genomic effects on the BK channels directly through estrogen receptor stimulation. However, the rapid increase in single BK channel *NP*_*o*_ by 17β-estradiol (Fig 3) clearly indicates that the BK channels are direct non-genomic targets for 17β-estradiol. As the role of estrogens in UBSM physiology and pathophysiology is not fully understood, the current study provides a foundational basis for future studies in human UBSM. Indeed, decreased estrogen levels have been associated with the increased occurrence of urinary urgency and frequency in post-menopausal women [3–5, 27]. Thus, the activation of the BK channels by 17β-estradiol, which in turn hyperpolarizes the membrane potential to cause UBSM relaxation, may underlie some of the observed benefits of estrogen replacement therapies in alleviating symptoms of OAB [5].

In conclusion, we reveal a new paradigm in UBSM cell physiology, indicating a direct role for the BK channels in mediating the inhibitory effects of 17β-estradiol on UBSM cell excitability.

## Abbreviations

BK channels: large conductance voltage- and Ca^2+^-activated K^+^ channels
Ca_V_ channels: L-type voltage-gated Ca^2+^ channels
DMSO: dimethyl sulfoxide
UBSM: urinary bladder smooth muscle
EGTA: ethylene glycol-bis(2-aminoethylether)-N,N,N',N'-tetraacetic acid
HEPES: 4-(2-Hydroxyethyl)piperazine-1-ethanesulfonic acid
*P_o_*: single channel open probability
*NP_o_*: single channel total open probability of each excised patch
OAB: overactive bladder
RyRs: ryanodine receptors
SR: sarcoplasmic reticulum
TBKCs: transient BK currents
